# Acute caffeine supplementation as a nutrition-based strategy to mitigate sleep-loss-related cognitive and operational performance impairments in military personnel: a systematic review and meta-analysis

**DOI:** 10.3389/fnut.2026.1893033

**Published:** 2026-09-04

**Authors:** Renchen Wang, Weifeng Wang, Lingwei Meng, Gang Hao, Guoxin Gu

**Affiliations:** Harbin Institute of Information Technology, Harbin, Heilongjiang, China

**Keywords:** caffeine, cognitive performance, military personnel, operational task performance, sleep deprivation, sleep restriction, vigilance

## Abstract

**Background:**

Sleep loss is common in military operations and degrades vigilance, higher cognitive function, and operational performance. Whether acute caffeine meaningfully attenuates these decrements in military settings has not been quantitatively synthesized.

**Methods:**

This study systematically searched PubMed, Web of Science, Cochrane Library, MEDLINE, Embase, and SPORTDiscus from database inception to the search cutoff date. Eligible studies were randomized controlled or crossover trials that included military personnel or military-related populations, administered caffeine under conditions of sleep deprivation, sleep restriction, or sustained wakefulness, and compared caffeine with placebo or a non-caffeinated control. The primary outcomes were categorized as vigilance and response performance, complex cognitive performance, and military task performance. Risk of bias was assessed using the RoB 2 tool, and meta-analyses were conducted using StataMP 17.0.

**Results:**

Seven randomized, double-blind, placebo-controlled studies were included. The meta-analysis showed that, compared with placebo, caffeine significantly improved vigilance and response performance under conditions of sleep deprivation or sleep restriction (SMD = 0.73, 95% CI: 0.50–0.96, I^2^ = 0.0%), complex cognitive performance (SMD = 0.54, 95% CI: 0.16–0.93, I^2^ = 0.0%), and military task performance (SMD = 0.45, 95% CI: 0.19–0.71, I^2^ = 29.2%). Overall, the ergogenic effects of caffeine were most consistent for vigilance and response performance, showed stable beneficial effects on complex cognitive performance, and were primarily reflected in improved task-execution speed and operational efficiency for military task performance.

**Conclusion:**

The available evidence suggests that acute caffeine supplementation improves vigilance and response performance during sleep loss in military personnel, with additional benefits for complex cognitive and military task performance. Effects were most consistent for vigilance and response speed, whereas benefits for complex cognition and military task performance were more task-dependent. These findings support caffeine as a practical short-term adjunct to fatigue management during military operations involving sleep loss, although further studies are needed to refine dose, timing, and task-specific use.

**Systematic review registration:**

https://www.crd.york.ac.uk/PROSPERO/view/CRD420261375742, identifier CRD420261375742.

## Graphical abstract

**Figure:**
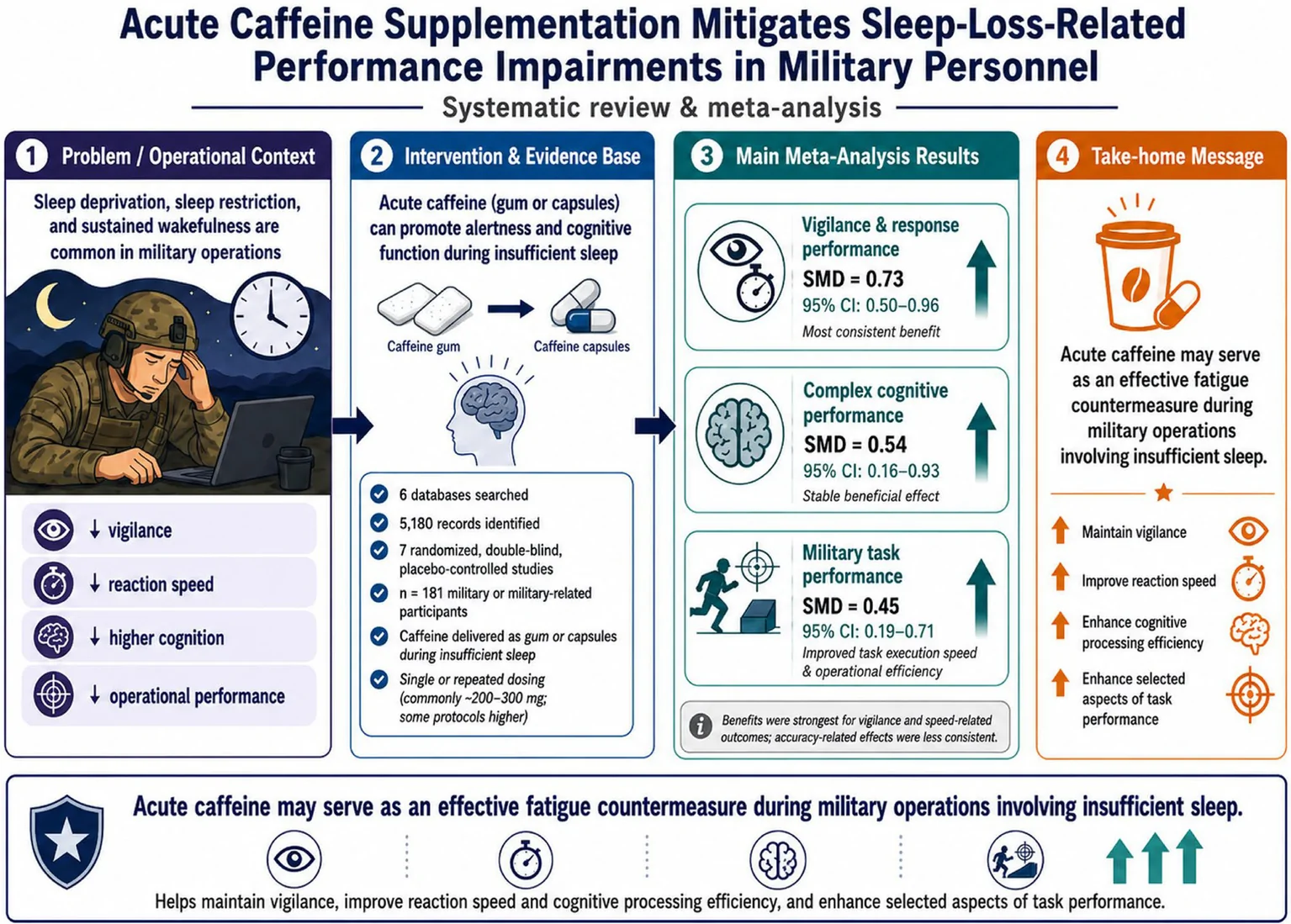

## 1 Introduction

Sleep insufficiency or sustained wakefulness commonly occurs during military operations and is known to substantially impair vigilance, reaction speed, executive function, and physical performance (1, 2). For example, during the U.S. Navy SEALs’ “Hell Week” training, severe sleep loss within only 72 h markedly reduced shooting accuracy and increased reaction time (2). Similar decrements in operational performance have also been frequently reported among army personnel and aviators. To maintain operational readiness, military personnel widely use stimulants, among which caffeine is the most common (3). By antagonizing adenosine receptors in the central nervous system and counteracting perceived fatigue, caffeine has been shown to improve attention and response capacity in both the general population and athletes (4).

Over the past two decades, multiple experimental studies have examined the ability of caffeine to attenuate the effects of sleep deprivation. Under normal or mildly fatigued conditions, caffeine has been shown to improve cognitive and physical performance (4, 5). Empirical studies conducted under extreme conditions have also provided important evidence. During Navy SEAL training involving approximately 72 h of wakefulness, 200–300 mg of caffeine significantly improved visual vigilance and reaction time without compromising shooting accuracy (6). Similarly, in Canadian military personnel, divided caffeine doses of 400 mg, 200 mg, and 100 mg restored the speed of hitting shooting targets, although shooting precision was not improved. In sustained-task scenarios, such as overnight special operations, timed oral administration of 200 mg caffeine capsules maintained daytime vigilance at a level close to that observed after normal sleep and reduced the completion time for obstacle-course and cross-country running tasks (7, 8). Collectively, these studies suggest that simple tasks, such as vigilance tests and reaction speed tasks, are often highly responsive to caffeine, whereas the effects on complex tasks, such as precision shooting or higher-order cognitive reasoning, are less pronounced (2, 9). In addition, repeated dosing, particularly continued nighttime administration, generally appears to be more effective than a single acute dose. However, systematic comparisons across different task types, dosing regimens, and participant characteristics, such as caffeine tolerance and circadian rhythm, remain limited (4, 8).

Although several original studies have been published, no comprehensive meta-analysis has yet examined the effects of caffeine intake on cognition and task execution in military personnel under sleep-deprived conditions. On the one hand, substantial variation across studies in participants, such as soldiers and pilots, settings, such as field training and simulated missions, and interventions, such as caffeine dose and delivery form, has produced inconsistent findings and made it difficult to draw unified conclusions (2, 9). On the other hand, previous reviews have largely focused on the general population or mixed samples, with insufficient attention to the high-stress and high-load tasks specific to military contexts (10, 11). We noted that a recent systematic review reported that caffeine may “improve cognitive, behavioral, and physiological performance” under sleep deprivation (1), but that review did not conduct stratified analyses by task category. Therefore, focusing specifically on military personnel and strictly following PRISMA and Cochrane methodological standards, the present study quantitatively evaluated the effect sizes and heterogeneity of caffeine across three domains: vigilance and response performance, higher-order cognitive function, and military task performance. Our aim was to determine whether and how caffeine can compensate for performance decrements induced by sleep deprivation, thereby providing quantitative evidence for fatigue management during military operations.

This study systematically synthesized data from relevant randomized controlled trials through meta-analysis. We sought to address the following specific questions: (1) Under conditions of sleep deprivation or sleep restriction, does caffeine significantly improve vigilance and response performance compared with placebo? (2) Does caffeine improve performance in complex cognitive tasks, such as logical reasoning, learning, and memory? (3) What effects does caffeine have on typical military task performance, such as shooting accuracy, operational speed, and completion time for physical tasks? Based on previous research, we hypothesized that caffeine would exert the most pronounced benefits on simple vigilance and speed-based tasks, whereas its effects would be limited for tasks such as precision shooting that depend on accuracy or higher-order processing.

## 2 Methods

### 2.1 Study registration

This study was conducted as a systematic review and meta-analysis. The study design and implementation followed the relevant methodological recommendations of the *Cochrane Handbook for Systematic Reviews of Interventions*, and the review was reported in accordance with the PRISMA reporting guidelines. The study protocol was prospectively registered in PROSPERO (registration number: CRD420261375742).

### 2.2 Search strategy

A systematic literature search was conducted in PubMed, Web of Science, Cochrane Library, MEDLINE, Embase, and SPORTDiscus. The search strategy combined controlled vocabulary terms and free-text terms, using “Caffeine” and “Sleep Deprivation” as core keywords, which were combined with Boolean operators (AND/OR). The complete search strategies for each database are provided in Supplementary Table S1. The search covered all records from database inception to the final search date, which was 15 March 2026. To ensure the accuracy and consistency of the search strategy, two researchers (R.W. and W.W.) independently checked the search terms and their logical combinations. Any disagreements were resolved through discussion with a third researcher (L.M.), who made the final decision.

### 2.3 Eligibility criteria

Eligibility criteria were defined according to the participants, intervention, comparator, outcomes, and study design framework. The inclusion criteria were as follows: Participants were military personnel or populations with a military-related background, including active-duty service members, special operations personnel, military academy students or trainees undergoing specialized training, and healthy adults tested in military-related task settings; the experimental intervention was caffeine supplementation, with no restriction on the form of administration, including but not limited to capsules and chewing gum; the study included a placebo-controlled or a non-caffeinated control condition; the study was conducted under conditions of sleep deprivation, partial sleep deprivation, sleep restriction, or sustained wakefulness; the study reported at least one outcome eligible for quantitative analysis, with outcomes primarily classified into three categories, namely, vigilance and response performance, higher-order cognitive performance, and task performance; the study provided data that could be extracted or used to calculate effect sizes, including sample size, mean, and standard deviation, or data that could be converted from information reported in the article; and the study used a randomized parallel-group, randomized crossover, or other controlled experimental design. A concurrent control condition was required to distinguish the effects of caffeine supplementation from performance changes attributable to sleep loss alone.

The exclusion criteria were as follows: Studies involving non-military general populations and not related to military tasks were excluded; studies that combined caffeine with other drugs, nutritional supplements, or interventions and did not allow the independent effect of caffeine to be distinguished were excluded; studies that examined only acute physiological responses, subjective perceptions, or outcomes unrelated to the objectives of the present study, without reporting vigilance and response performance, higher-order cognitive performance, or task performance, were excluded; studies without a control condition, as well as studies for which key data such as sample size, mean values, and measures of dispersion could not be obtained and could not be included in effect-size calculation after supplementary data retrieval, were excluded; reviews, conference abstracts, case reports, commentaries, and animal studies were also excluded.

### 2.4 Study selection and data extraction

All retrieved records were imported into Zotero. Titles and abstracts were screened to identify potentially eligible studies. The full texts of these studies were then downloaded and assessed to determine the final set of original studies that met the inclusion criteria. Before data extraction, a standardized data extraction form was developed. The extracted information included study identification information, study design, participants and setting, sample size and sex distribution, age, caffeine intervention and comparator, sleep-loss model, and outcomes included in the meta-analysis. All continuous outcome data used for effect-size calculation were extracted in the form of mean ± SD whenever available. When study results were presented graphically, data were extracted using WebPlotDigitizer 4.6 (Pacifica, California, USA) (*r* = 0.99, *p* < 0.001) (12). Study selection and data extraction were independently performed by two researchers (R.W. and W.W.) and cross-checked after completion. Disagreements during data extraction were resolved by consensus or, when necessary, through consultation with a third researcher (L.M.).

### 2.5 Risk-of-bias assessment

The methodological quality of all included studies was assessed using the Cochrane risk-of-bias assessment tool. The risk of bias was classified as “low risk,” “some concerns,” or “high risk.” The assessment results were used to evaluate the overall quality of the included studies and served as an important basis for subsequent interpretation of the evidence. Risk-of-bias assessment was independently performed and cross-checked by two researchers (R.W. and W.W.). Any disagreements were resolved through discussion, and, when necessary, a third researcher (L.M.) was consulted for adjudication. Publication bias was assessed using funnel plots in combination with Egger’s regression test and Begg’s rank correlation test. When potential publication bias was indicated, the trim-and-fill method was further applied for correction and sensitivity assessment. To further examine the robustness of the main pooled results, sensitivity analyses were also performed using the leave-one-out method, in which each study was sequentially removed to determine whether the pooled effect size and its confidence interval changed substantially after the exclusion of any individual study.

### 2.6 Statistical analysis

All statistical analyses were performed using StataMP 17.0 (Stata 17). Because all outcomes included in the present study were continuous variables, outcome data were expressed as means and standard deviations. Effect sizes were calculated using the standardized mean difference (SMD), and forest plots were generated accordingly. To account for potential clinical and methodological differences across studies in intervention protocols, participant characteristics, and testing methods, pooled effect sizes were calculated using a random-effects model regardless of the magnitude of heterogeneity (13, 14). Heterogeneity was assessed using the I^2^ statistic and interpreted as follows: I^2^ < 25% indicated low heterogeneity, 25–75% indicated moderate heterogeneity, and I^2^ > 75% indicated high heterogeneity. The magnitude of the effect size was interpreted according to the classification criteria proposed by Hopkins et al. (15): SMD < 0.2 was considered a trivial effect, 0.2–0.6 a small effect, 0.6–1.2 a moderate effect, 1.2–2.0 a large effect, 2.0–4.0 a very large effect, and >4.0 an extremely large effect. Publication bias was visually assessed using funnel plots and statistically examined using Egger’s regression test and Begg’s rank correlation test (16). When potential publication bias was indicated, the trim-and-fill method was further applied for adjusted estimation. Sensitivity analysis was conducted to examine the robustness of the pooled results using a leave-one-out approach, in which one study was removed at a time and the effect size was recalculated to evaluate the influence of each individual study on the overall conclusions. All tests were two-sided, and the level of statistical significance was set at *p* < 0.05.

## 3 Results

### 3.1 Search results

**Figure 1:**
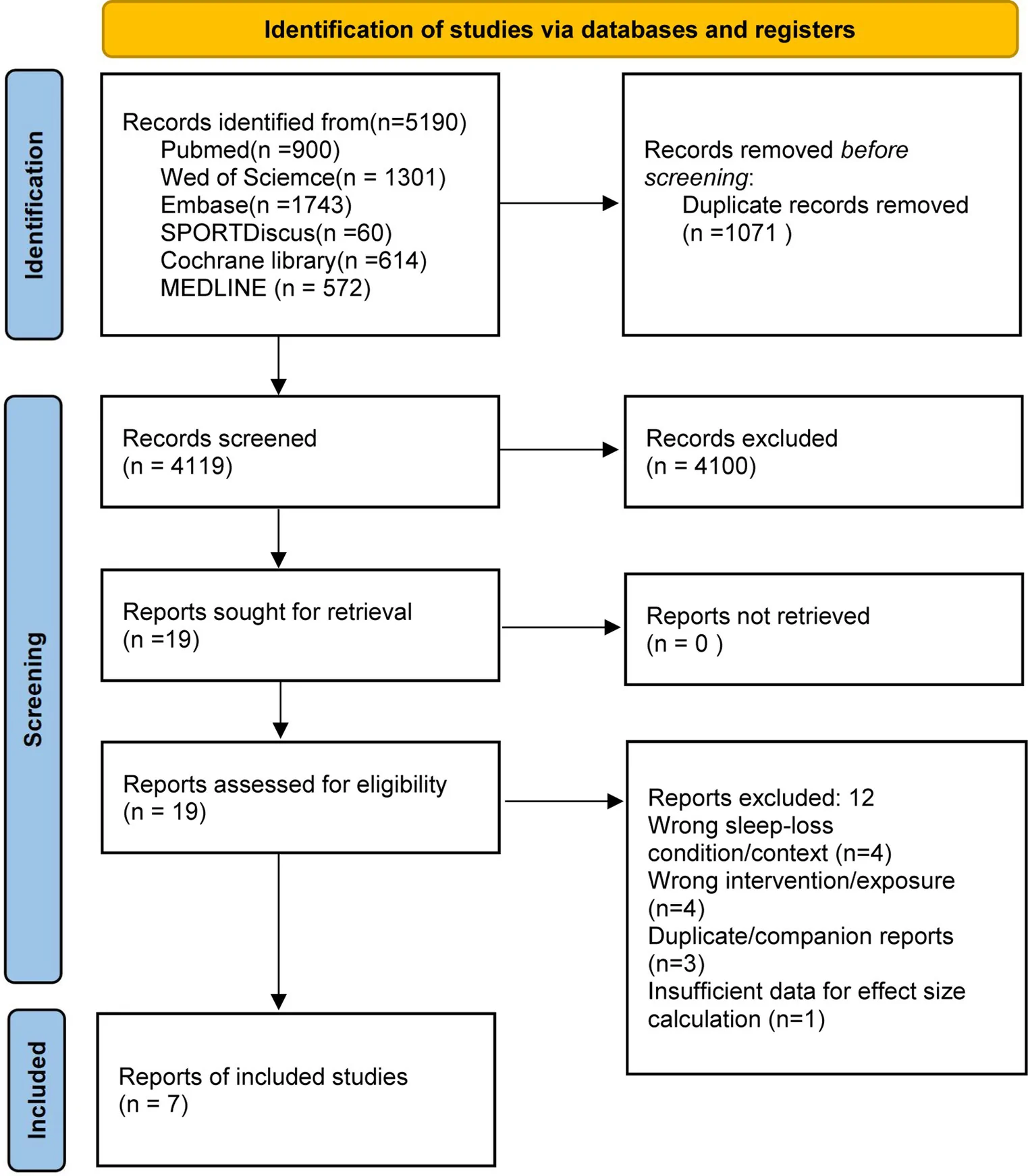
Flowchart of screening process.

A total of 5,190 records were retrieved from the databases. After screening according to the inclusion and exclusion criteria, seven studies were ultimately included in the meta-analysis. The study selection process is shown in Figure 1. Detailed search strategies are provided in Supplementary Appendix 1, and full-text articles excluded after eligibility assessment, together with the primary reasons for exclusion, are listed in Supplementary Appendix 2.

### 3.2 Study characteristics

Seven studies published between 2002 and 2023 were included. All included studies adopted randomized, double-blind, placebo-controlled designs, comprising five parallel-group trials and two crossover trials. The study populations were primarily military personnel or military-related populations, including special operations personnel, naval special warfare trainees, rifle-trained military personnel, and air force personnel. Except for the study by Wingelaar-Jagt et al., which included female participants, all other studies included only male participants. The study settings covered military or military-like operational conditions, including total sleep deprivation, sleep restriction, and sustained wakefulness. Caffeine was mainly administered in the form of chewing gum or capsules. The outcomes included in the quantitative synthesis primarily involved vigilance and response performance, higher-order cognitive performance, and task performance. Further details are presented in Table 1.

**Table 1:** Characteristics of the included studies.

Study	Design	Participants/setting	N (M/F)	Age, years	Intervention/comparator	Sleep-loss model	Outcomes in meta-analysis
McLellan et al. (2007) (8)	Randomized, double-blind, parallel placebo-controlled trial	Male Special Forces personnel, multinational military setting	20 (20/0)	28.6 ± 4.7	Caffeine gum, 200 mg at 21:45 on Days 2–4 and 01:00, 03:45, 07:00 on Days 3–5 versus (vs.) matched placebo gum	4 d/3 nights sustained operations with overnight wakefulness; 4-h daytime sleep on Days 3–4	Vigilance score; total course time; obstacle time; run time
Tikuisis et al. (2004)	Double-blind, placebo-controlled crossover trial	Rifle-trained military men, Canada	20 (20/0)	26.7 ± 7.2	Caffeine gum, 400 + 100 + 100 mg at 7.5, 3, and 0 h before shooting vs. placebo gum	24-h control period including sleep, followed by 22 h sustained wakefulness	Target engagement time; number of foe shots fired; number of foe targets hit
McLellan et al. (2005) (7)	Randomized, double-blind, parallel placebo-controlled trial	Male Special Forces personnel, multinational military setting	31 (31/0)	29.9 ± 5.3	Caffeine gum, 200 mg at 02:00, 04:00, and 06:00 vs. placebo gum	Single overnight field operation with sleep deprivation	ORVT vigilance score; PVT number of lapses; 6.3-km run time; marksmanship accuracy
Lieberman et al. (2002) (4)	Randomized, double-blind, placebo-controlled parallel-group trial	U. S. Navy SEAL trainees, United States	28 (28/0)^*^	23.9 ± 3.0	300 mg caffeine capsule, single dose vs. placebo capsule	~72 h sleep deprivation during SEAL Hell Week with severe environmental and operational stress	Scanning visual vigilance hits; scanning visual vigilance response time; four-choice visual reaction time correct hits; repeated acquisition time-to-completion
Wingelaar-Jagt et al. (2023) (34)	Randomized, double-blind, placebo-controlled crossover trial	Royal Netherlands Air Force employees, Netherlands	32 (27/5)	35 ± 10	300 mg caffeine capsule, single dose at midnight vs. placebo capsule	Limited overnight wakefulness; median wake time at dosing 17 h; testing until 08:00	PVT number of lapses
Kamimori et al. (2015) (35)	Randomized, double-blind, parallel placebo-controlled trial	Male Special Forces personnel, multinational military setting	20 (20/0)	28.6 ± 4.7	Caffeine gum, four 200-mg doses per 24 h at 21:45, 01:00, 03:45, and 07:00 vs. placebo gum	Three successive nights of sustained wakefulness with 4-h afternoon sleep opportunities	PVT speed; FVT score; vigilance monitor correct responses; Logical Reasoning Test correct response time
Tharion et al. (2003) (2)	Randomized, double-blind, placebo-controlled parallel-group trial	Navy SEAL trainees, United States	30 (30/0)^*^	24 ± 0.3^†^	200 mg caffeine capsule, single dose vs. placebo capsule	~72 h total sleep deprivation during SEAL Hell Week with cold-wet and operational stress	Distance from center of mass; shot group tightness; sighting time; percent of targets missed

^*^Sample size shown for the comparison included in the quantitative synthesis; the original trials enrolled more treatment arms. ^†^Reported as mean ± SEM in the original article. ORVT, Observation and Reconnaissance Vigilance Task; PVT, Psychomotor Vigilance Task; FVT, Field Vigilance Test; SEAL, Sea, Air, and Land; M/F, male/female.

### 3.3 Risk-of-bias assessment of the included studies

**Figure 2:**
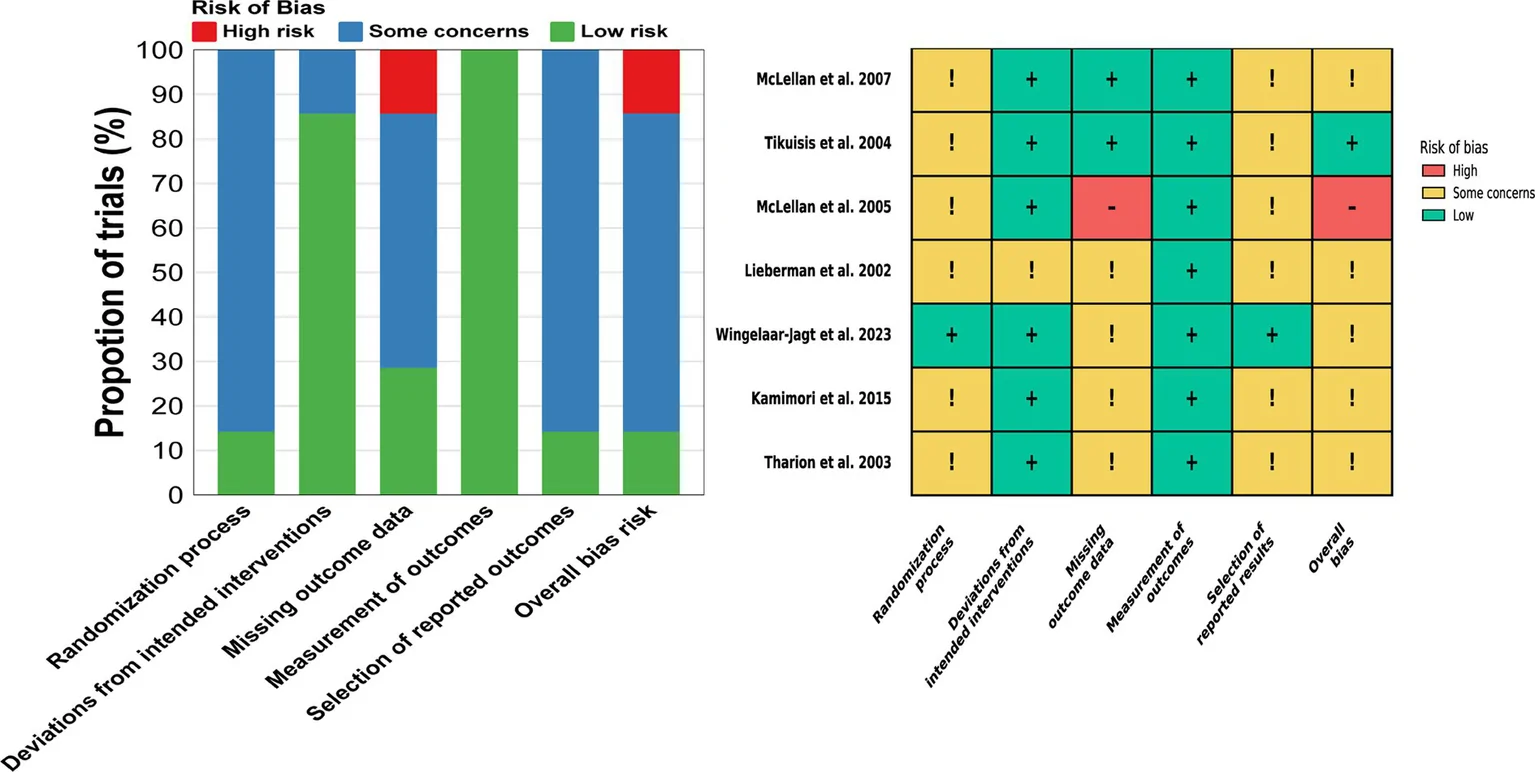
Bias risk map.

The methodological quality of the seven included studies was assessed using the Cochrane risk-of-bias tool (RoB 2). The assessment domains included the randomization process, deviations from intended interventions, missing outcome data, outcome measurement, and selective reporting. The detailed results are presented in Figure 2.

Overall, the methodological quality of the included studies was generally acceptable; however, most studies raised some concerns in specific domains. For the randomization process, one study was rated as low risk, whereas six studies were rated as having some concerns. For deviations from intended interventions, six studies were rated as low risk and one study as having some concerns. For missing outcome data, two studies were rated as low risk, four studies as having some concerns, and one study as high risk. For outcome measurement, all studies were rated as low risk. For selective reporting, one study was rated as low risk, whereas the remaining six studies were rated as having some concerns.

In the overall risk-of-bias judgment, one study was rated as low risk, one study as high risk, and the remaining five studies as having some concerns. In general, the main methodological limitations of the included studies were insufficient reporting of the randomization process, potential risk associated with missing outcome data in some studies, and inadequate information on selective reporting. By contrast, the risk of bias related to outcome measurement was relatively low.

### 3.4 Meta-analysis results

#### 3.4.1 Vigilance and response performance

**Figure 3:**
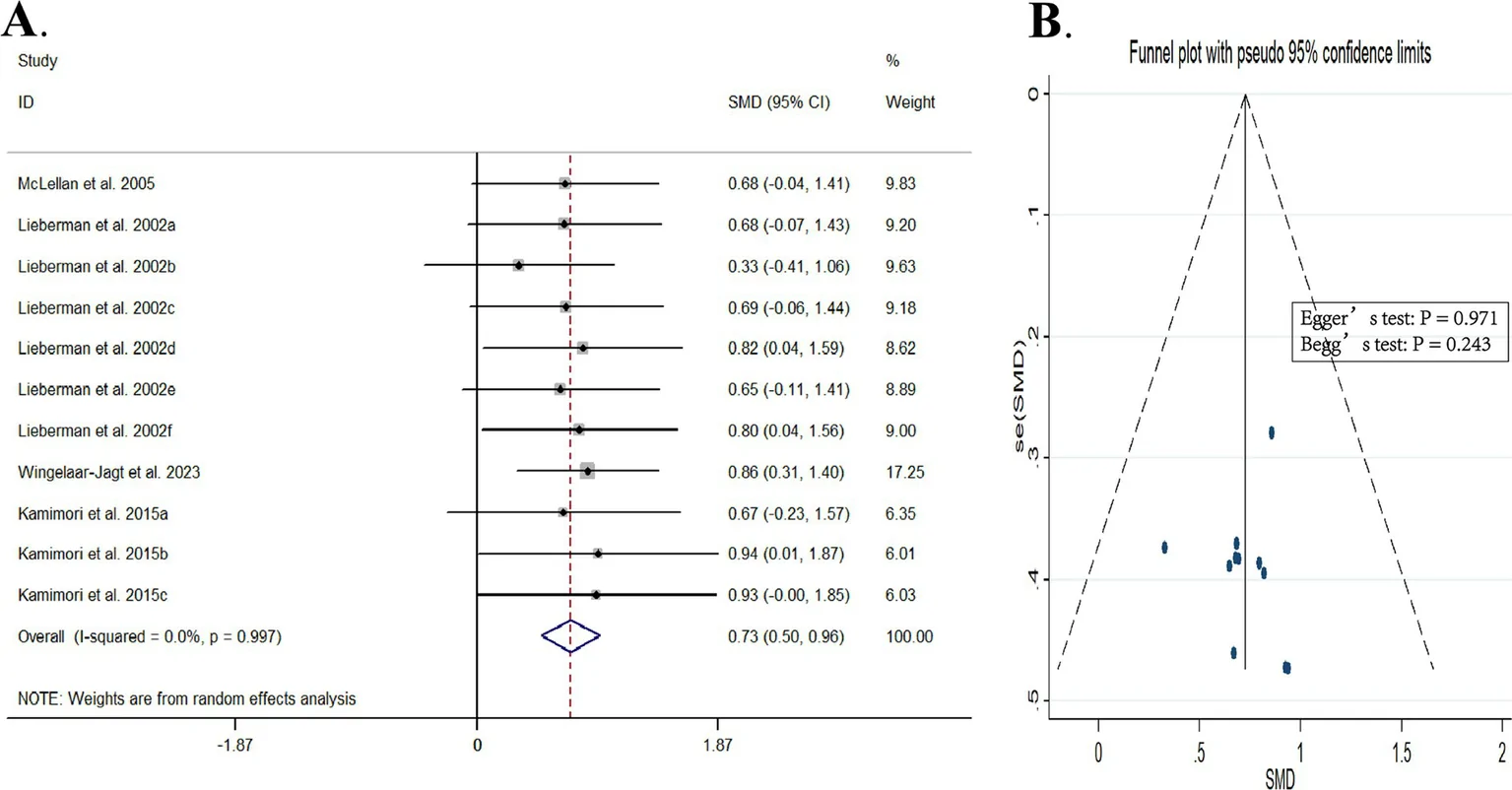
Pooled effect of caffeine on vigilance and response performance and assessment of publication bias. **(A)** Forest plot; **(B)** Funnel plot, with Begg’s and Egger’s tests.

The pooled analysis of vigilance and response performance showed that caffeine had a statistically significant beneficial effect on vigilance and response performance under conditions of sleep deprivation or sleep restriction, with a moderate effect size (SMD = 0.73, 95% CI: 0.50–0.96; Figure 3A). This finding suggests that caffeine helps improve this category of outcomes compared with placebo.

The heterogeneity analysis indicated a high degree of consistency across studies, with extremely low heterogeneity (I^2^ = 0.0%, *p* = 0.997), suggesting that the direction and magnitude of the effects on vigilance and response performance were generally consistent among the included studies. The relative weights of individual effect sizes ranged from 6.01 to 17.25%. Publication bias analysis showed that the funnel plot was generally symmetrical, with no obvious asymmetry (Figure 3B). Begg’s rank correlation test yielded *p* = 0.243, and Egger’s regression test yielded *p* = 0.971, neither of which indicated significant small-study effects or obvious publication bias.

#### 3.4.2 Complex cognitive performance

**Figure 4:**
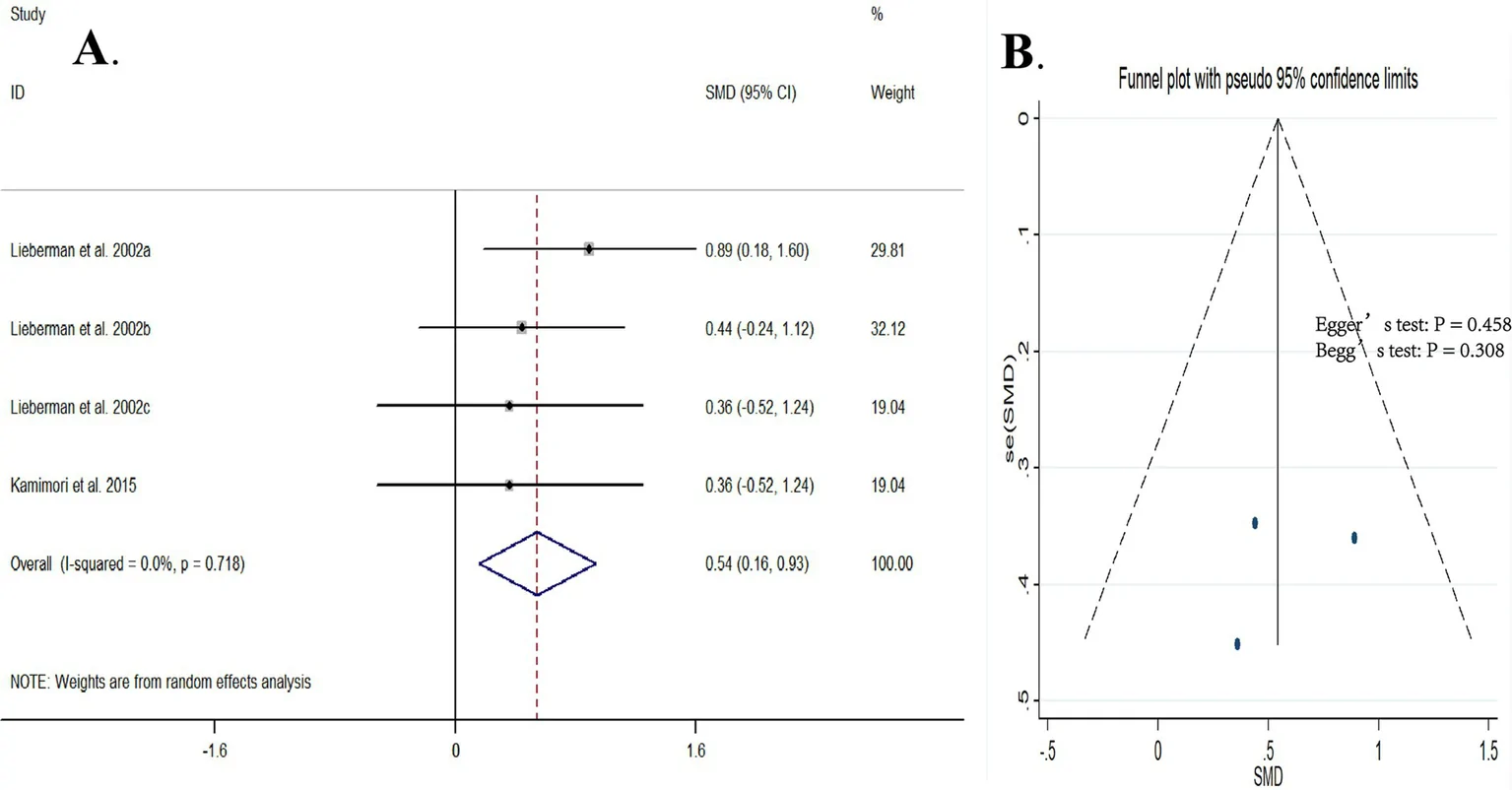
Pooled effect of caffeine on complex cognitive performance and assessment of publication bias. **(A)** Forest plot; **(B)** Funnel plot, with Begg’s and Egger’s tests.

Outcomes related to complex cognitive performance were primarily derived from studies examining logical reasoning, learning and memory, and complex reaction tasks under military sleep-deprivation conditions. The meta-analysis showed that caffeine had a statistically significant beneficial effect on complex cognitive performance under conditions of sleep deprivation or sleep restriction, with an overall small-to-moderate effect size (SMD = 0.54, 95% CI: 0.16–0.93; Figure 4A). This finding suggests that, compared with placebo, caffeine contributes to improved performance in this category of outcomes.

The heterogeneity analysis indicated a high degree of consistency across studies, with extremely low heterogeneity (I^2^ = 0.0%, *p* = 0.718), suggesting that the direction and magnitude of the effects on complex cognitive performance were generally consistent among the included studies. Overall, caffeine demonstrated relatively consistent beneficial effects on complex cognitive processing speed, logical reasoning efficiency, and related task performance. Publication bias analysis showed that the funnel plot was generally symmetrical, with no obvious asymmetry (Figure 4B). Begg’s rank correlation test yielded *p* = 0.308, and Egger’s regression test yielded *p* = 0.458, neither of which indicated significant small-study effects or obvious publication bias. However, because the number of studies included in this analysis was relatively small, these results should still be interpreted with caution.

#### 3.4.3 Military task performance

**Figure 5:**
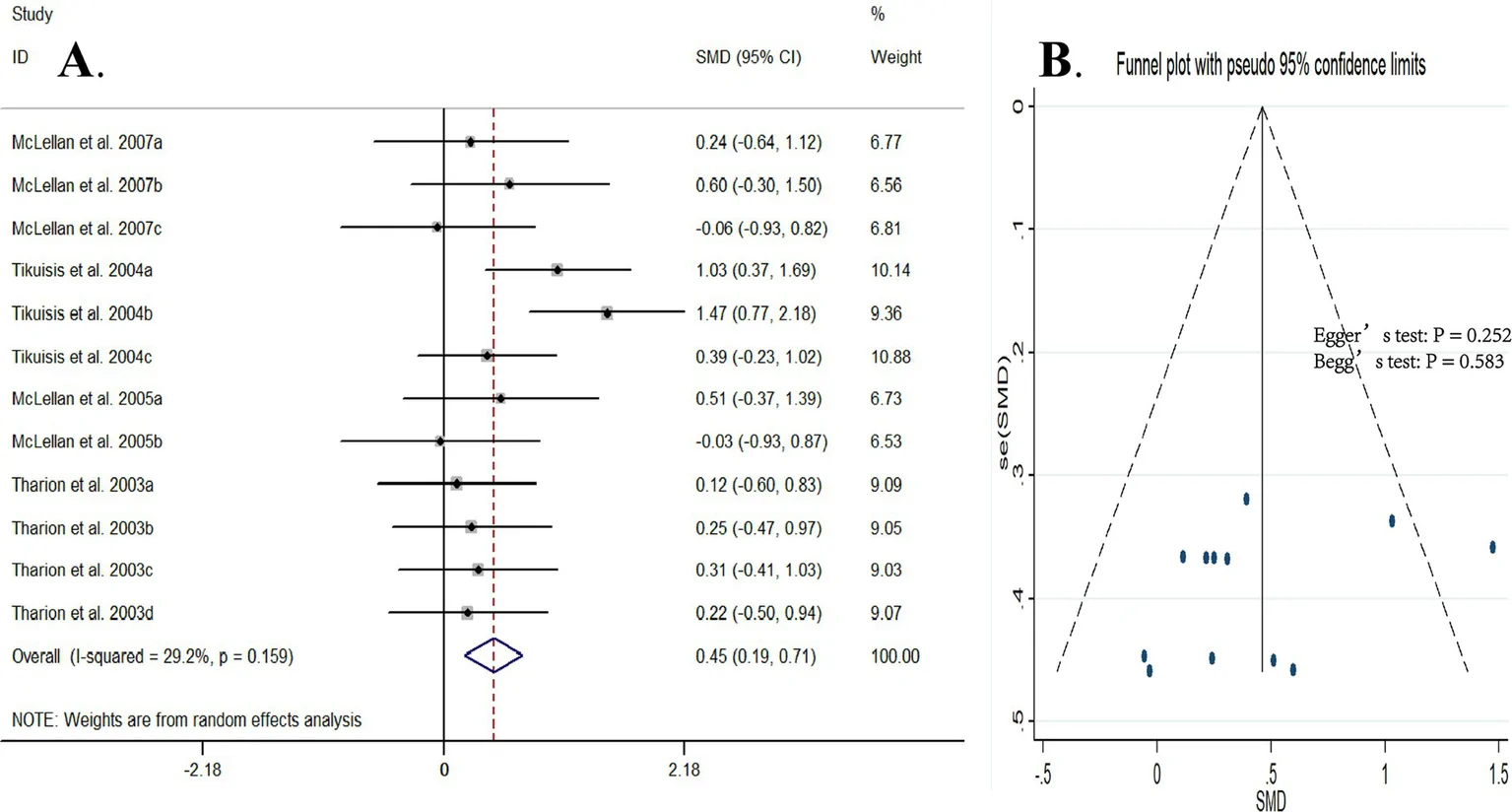
Pooled effect of caffeine on military task performance and assessment of publication bias: **(A)** Forest plot; **(B)** Funnel plot, with Begg’s and Egger’s tests.

Outcomes related to military task performance were primarily derived from studies examining obstacle-course performance, running performance, target engagement, shooting speed, and shooting accuracy under conditions of sleep deprivation or sustained wakefulness. The meta-analysis showed that caffeine had a statistically significant beneficial effect on military task performance under conditions of sleep deprivation or sleep restriction, with an overall small-to-moderate effect size (SMD = 0.45, 95% CI: 0.19–0.71; Figure 5A). This finding suggests that, compared with placebo, caffeine contributes to improved performance in this category of outcomes.

The heterogeneity analysis indicated generally good consistency across studies, with low heterogeneity (I^2^ = 29.2%, *p* = 0.159), suggesting that the direction of the effects on military task performance was broadly consistent across studies, although some variation in effect magnitude was observed. Overall, caffeine demonstrated relatively stable beneficial effects on task-execution speed, operational efficiency, and selected aspects of military task performance. Publication bias analysis showed that the funnel plot was generally symmetrical, with no obvious asymmetry (Figure 5B). Begg’s rank correlation test yielded *p* = 0.583, and Egger’s regression test yielded *p* = 0.252, neither of which indicated significant small-study effects or obvious publication bias.

### 3.5 Sensitivity analysis

To examine the robustness of the pooled results, leave-one-out sensitivity analyses were performed by sequentially excluding each individual effect estimate. The results are presented in Supplementary Figures S3.1–S3.3. For vigilance and response performance (Supplementary Figure S3.1), the pooled effect remained positive after the omission of each individual effect estimate, with only minor changes in effect magnitude and no substantive change in statistical inference, indicating good robustness of the primary analysis. For complex cognitive performance (Supplementary Figure S3.2), the direction of the pooled effect remained consistent after each individual effect estimate was excluded. Although the overall effect estimate showed some fluctuation, the conclusion that caffeine favored performance remained unchanged, suggesting that the pooled result for this outcome was generally stable. For military task performance (Supplementary Figure S3.3), the pooled effect also remained positive after the omission of each individual effect estimate, with only limited changes in effect magnitude and consistent statistical inference. Overall, the pooled results across all three outcome domains were not disproportionately influenced by any individual effect estimate, supporting the robustness of the findings.

## 4 Discussion

This meta-analysis is the first to systematically evaluate the effects of caffeine intervention in sleep-deprived populations within military-related contexts. The results showed that, compared with placebo, caffeine significantly improved multiple aspects of performance in military personnel. A moderate effect was observed for vigilance and response performance (SMD ≈ 0.73, 95% CI: 0.50–0.96), suggesting that caffeine can consistently improve vigilance and simple reaction speed after nighttime duty or prolonged wakefulness. A small-to-moderate effect was also observed for complex cognitive performance (SMD ≈ 0.54, 95% CI: 0.16–0.93), indicating that caffeine may provide some benefit for higher-order cognitive tasks, such as logical reasoning and information processing. The pooled effect size for military task performance was 0.45 (95% CI: 0.19–0.71), suggesting that caffeine can, to some extent, enhance the speed and efficiency of tactical actions, such as obstacle-course performance and target engagement. Overall, heterogeneity across studies was low for all outcome domains (vigilance: I^2^ ≈ 0; complex cognition: I^2^ ≈ 0; task performance: I^2^ ≈ 29%), and sensitivity analyses showed that the overall effects remained stable after the removal of any single study, supporting the reliability of the findings. These results indicate that caffeine, as an adenosine receptor antagonist, can partially offset sleep deprivation-induced declines in vigilance and cognitive function, which is consistent with its established clinical use for enhancing alertness (10).

Compared to previous studies, the present findings are more convergent than divergent. A focused military review concluded that caffeinated products can preserve cognitive, behavioral, and physical functioning during sleep deprivation in military personnel (1), while a broader meta-analysis in sleep-restricted or sleep-deprived adults reported beneficial effects of acute caffeine consumption across attention, information processing, executive function, reaction time, and driving-related outcomes (10). More broadly, McLellan et al. (17) reported that low-to-moderate caffeine doses reliably enhance alertness, vigilance, attention, and reaction time, whereas effects on memory and higher-order executive processes are less uniform. Against this background, the present finding that the clearest and most consistent benefits were observed for vigilance and response performance, with additional but more task-dependent improvements in complex cognitive and military task performance, is aligned with the wider literature. The principal difference is therefore not the direction of effect, but the greater variability of military task outcomes, which likely reflects the heterogeneous combination of speed, precision, decision-making, and motor demands inherent to operational tasks.

Comparisons with non-military occupational groups point to a similar core pattern. In fatigued shift workers relevant to emergency medical services, Temple et al. (2018) (18) reported that caffeine improved psychomotor vigilance and end-of-shift reaction time, and the Cochrane review by Ker et al. (2021) (19) likewise concluded that caffeine can reduce errors and improve cognitive performance in shift workers, even though evidence for downstream injury outcomes remains unavailable. These findings closely mirror the present review in suggesting that caffeine most consistently preserves vigilance and psychomotor speed, whereas translation to broader safety or operational outcomes is less directly demonstrated. At the same time, the literature in healthcare workers and firefighters differs importantly in design. A recent systematic review in shift-working healthcare professionals found that caffeine was the most pervasive fatigue-coping strategy under night and rotating schedules, but the evidence was predominantly observational rather than based on randomized performance trials (20). Similarly, longitudinal firefighter data indicate that associations between caffeine use and sleep are dynamic and person-dependent, rather than uniformly beneficial or harmful (21). Taken together, these comparisons suggest that while reliance on caffeine under sleep loss is shared across safety-critical occupations, a military-specific meta-analysis remains necessary because military studies more often model acute operational sleep loss under controlled conditions and evaluate mission-relevant performance endpoints that are not captured by most civilian shift-work studies.

It should be noted that the present study focused specifically on military task contexts, with outcomes including combat-relevant tasks such as shooting and obstacle-course performance, which have been less frequently addressed in previous reviews. Previous studies focused on written tests or computer-based tasks, whereas the studies included in this review also assessed tactical simulation tasks, such as the Tactical Combat Movement Simulation. The results showed that although caffeine generally had a beneficial effect on these tasks, the effect size was relatively small. This finding is consistent with the results reported by Stein et al. in a simulated combat sprint task, in which 4 mg/kg caffeine chewing gum did not improve shooting accuracy, sprint duration, or cognitive performance during the task (6). Similarly, Clemente-Suarez et al. found that ingestion of 400 mg caffeine increased cognitive and somatic anxiety in soldiers but did not improve shooting performance during close-quarter combat (22). These findings suggest that for complex tasks involving fine motor control or extreme physical load, caffeine may increase subjective arousal and physiological activation but may not directly translate into performance gains.

Caffeine exerts its effects primarily by antagonizing adenosine receptors in the central nervous system (10, 17). Sleep deprivation leads to the accumulation of adenosine in the brain, thereby increasing sleep pressure and impairing vigilance and cognitive function (10, 23). By occupying A1 and A2A receptors, caffeine attenuates the inhibitory effects of adenosine and enhances the activity of arousal-related neurotransmitters, such as dopamine and norepinephrine, thereby improving vigilance, attention, and motivational state (3, 24). This mechanism helps explain why basic attention and reaction-time tasks benefited most clearly: These tasks are primarily governed by sustained vigilance and attentional state, and caffeine can effectively compensate for the attentional decline caused by insufficient sleep (25). Our data also showed that simple reaction tasks, such as the psychomotor vigilance task and vigilance tests, had the largest effect sizes among the included outcomes, which is consistent with this mechanism.

By contrast, higher-order cognitive tasks, such as logical reasoning, learning, and memory, require not only vigilance but also resources such as working memory capacity and executive control (17). Evidence suggests that the effects of sleep deprivation on these complex cognitive processes cannot be fully explained by reduced vigilance alone and may also involve direct impairment of specific functions such as problem-solving or memory encoding (17, 25). The review by Hudson et al. indicated that although sustained vigilance is an important foundation for many cognitive tasks, individual susceptibility to sleep loss can dissociate between vigilant attention and other cognitive processes, suggesting that sleep deprivation does not affect complex cognitive performance solely through reduced vigilance (26). A recent meta-analysis on sleep loss and executive function also showed that insufficient sleep broadly impairs core executive functions, including working memory, inhibitory control, and cognitive flexibility, and that the impairment patterns differ across task components (27). Stepan et al. found in a placekeeping task that caffeine significantly improved only psychomotor vigilance performance, whereas its effects on higher-order procedural memory were limited (25). This is similar to the pattern observed in the present study: Although the effect on complex cognitive performance reached statistical significance, its magnitude was clearly smaller than that for vigilance. This may indicate that caffeine has a limited restorative effect on the vigilance system and cannot fully reverse specific executive function deficits caused by insufficient sleep.

In addition, the effects of caffeine on military task performance are closely related to task characteristics. For example, in contexts requiring fine motor skills or endurance, caffeine may shorten task completion time by improving vigilance and fatigue tolerance, but it appears to provide limited benefit for movement precision (3, 6). The military tasks included in the present analysis mainly involved running speed, shooting reaction time, and overall operational efficiency, all of which are partly influenced by vigilance. Therefore, the overall effect observed in this analysis favored caffeine. However, in real-world operational contexts, factors such as load carriage, environmental stress, and continuous task demands may attenuate the effects of caffeine. For example, Stein et al. found that even when arousal was increased, sprint duration and defensive responses under heavy load were not improved, suggesting that physiological fatigue or skill-related limitations may impose stronger constraints on operational performance (6).

The efficacy of caffeine is also moderated by dose and individual characteristics. Irwin et al., using meta-regression, found that caffeine dose explained part of the variation in effects, with positive effects observed across doses ranging from <80 mg to 600 mg; however, different tasks varied in their dose sensitivity (10). In the present study, most included caffeine doses ranged from 200 to 400 mg, broadly consistent with the effective dose range recommended in the literature, approximately 3–6 mg/kg (10, 17). However, caffeine-use habits were insufficiently controlled in the included studies, and habitual users often show attenuated responses to caffeine (28). A report by the Institute of Medicine noted that chronic caffeine intake may induce upregulation of adenosine receptors, meaning that habitual users may require higher doses to obtain comparable performance-enhancing effects (28). Therefore, individual tolerance and prior caffeine exposure may influence the magnitude of the pooled effects and should be considered when interpreting the findings. In addition, caffeine-induced changes in hormonal responses, such as epinephrine, and physiological function, such as heart rate and blood lactate, may also influence task performance.

The findings of this study have important implications for military and operational applications. The evidence suggests that, during sustained operations, prolonged watch duty, or shift work, moderate caffeine intake can serve as an effective acute countermeasure against declines in vigilance and selected aspects of cognitive function (10, 17). For example, the U.S. military has recommended 200–300 mg caffeine as a common dose during nighttime duty or extreme training to maintain basic attention and reaction capacity, as indicated in the report of the U.S. National Academies (29). However, caffeine is not a universal solution and cannot fully eliminate the negative consequences of insufficient sleep. Operators should follow the principle of appropriate dosing, avoid excessive intake that may induce adverse effects such as anxiety and increased heart rate, and combine caffeine with other strategies, such as brief naps and task rotation, to improve operational efficiency. External validity should nevertheless be considered carefully. Most participants in the present review were male and were drawn from special operations or similarly selected military cohorts. This is important because caffeine-use patterns vary across sex, service branch, and occupational specialty in the wider active-duty force (30) and because special operations personnel may differ from conventional military populations in training background, baseline fitness, habitual stimulant use, and tolerance of demanding sleep-loss conditions. Recent data from Special Operations Forces further suggest that sleep-performance relationships in this population may not mirror those observed in civilian samples, reinforcing the possibility that findings from highly selected military groups are not automatically generalizable to the broader force (31). Generalizability to female service members is also uncertain. In active-duty U.S. Navy sailors, Shattuck et al. (2025) (32) identified meaningful sex differences in daytime sleepiness, psychomotor vigilance performance, and patterns of caffeinated beverage use in an operational setting. Accordingly, the present findings are most directly applicable to male, highly trained military personnel exposed to acute sleep loss, and further trials should deliberately recruit women and conventional-force populations.

This study also has several limitations. First, only seven randomized controlled trials with a relatively small overall sample were included, limiting the precision of the pooled estimates and the ability to examine potential moderators. The small number of studies also limited the statistical power of Begg’s and Egger’s tests to detect funnel plot asymmetry. Therefore, their non-significant results should be interpreted as an absence of detected small-study effects rather than evidence that publication bias was absent (33). Second, the included studies differed in their task types and cognitive outcomes, and specific experimental conditions, such as the type and duration of sleep deprivation and timing of caffeine intake, were not fully consistent, which may have introduced unmeasured sources of heterogeneity. Third, individual differences, including habitual caffeine intake and genetic polymorphisms such as CYP1A2, were not controlled and may affect the interpretation of the results. Methodologically, we did not distinguish between caffeine administration forms, such as capsules and chewing gum, or between time-dependent effects, although previous studies have suggested that these factors may also moderate the magnitude of caffeine’s effects. Finally, this meta-analysis focused on short-term intervention effects, and it remains unclear whether the same effects would occur with prolonged continuous use or repeated use across multiple days. Future research should further examine different dosing strategies, genetic and sex-related differences, complex task performance, and the potential synergistic effects of caffeine combined with other countermeasures, such as light exposure and short rest periods.

In summary, the present study indicates that moderate caffeine intake under conditions of sleep deprivation or sleep restriction significantly improves vigilance, basic reaction speed, and general cognitive function in military personnel and may also produce beneficial effects on selected aspects of tactical task performance. The primary mechanism of caffeine involves antagonizing sleep deprivation-induced adenosine-mediated inhibition and thereby increasing central nervous system arousal (17). However, caffeine cannot fully counteract all functional impairments caused by insufficient sleep, and its effects appear relatively limited for more complex cognitive tasks and fine motor control (2). Therefore, in practical applications, caffeine dose and timing should be carefully balanced, and caffeine should be used as one component of a broader fatigue management strategy rather than as a stand-alone solution. Future studies should continue to optimize individualized caffeine intervention protocols and clarify the boundaries of its effects across different task types and physiological profiles, thereby providing stronger scientific guidance for military operations, night-shift work, and emergency response.

## 5 Conclusion

Our findings suggest that acute caffeine supplementation attenuates performance decrements associated with sleep loss in military personnel. The clearest and most consistent benefits were observed for vigilance and response performance, while meaningful improvements were also evident in complex cognitive and military task performance. The observed effects were most apparent for sustained attention, response speed, cognitive processing efficiency, and task-execution speed, although effects on precision-dependent outcomes, including marksmanship accuracy, were less consistent.

Taken together, these findings support acute caffeine supplementation as a practical short-term component of fatigue management during military operations involving restricted sleep or prolonged wakefulness. The limited number of studies and participants constrains the precision and generalizability of the effect estimates. Further research is needed to refine dose and timing strategies and to determine whether these findings extend across a broader range of military personnel and operational settings.

## Data Availability

The original contributions presented in the study are included in the article/Supplementary material, further inquiries can be directed to the corresponding author.

## References

[1] Chaudhary NS Taylor BV Grandner MA Troxel WM Chakravorty S. The effects of caffeinated products on sleep and functioning in the military population: a focused review. Pharmacol Biochem Behav. (2021) 206:173206. doi: 10.1016/j.pbb.2021.173206, 34000324 PMC8487254

[2] Tharion WJ Shukitt-Hale B Lieberman HR. Caffeine effects on marksmanship during high-stress military training with 72 hour sleep deprivation. Aviat Space Environ Med. (2003) 74:309–14. 12688447

[3] Gillingham R Keefe A Tikuisis P. Acute caffeine intake before and after fatiguing exercise improves target shooting engagement time. Aviat Space Environ Med. (2004) 75:865–71. 15497366

[4] Lieberman H Tharion W Shukitt-Hale B Speckman K Tulley R. Effects of caffeine, sleep loss, and stress on cognitive performance and mood during U.S. navy SEAL training. Sea-air-land. Psychopharmacology. (2002) 164:250–61. doi: 10.1007/s00213-002-1217-9, 12424548

[5] Jovanovic M Sporis G Sopar J Harasin D Matika D. The effects of basic military training on shooting tasks in conditions of sleep deprivation. Kinesiology. (2012) 44:31–8.

[6] Stein JA Hepler TC DeBlauw JA Beattie CM Beshirs CD Holte KM . Caffeine gum does not improve marksmanship, bound duration, susceptibility to enemy fire, or cognitive performance during tactical combat movement simulation. J Spec Oper Med. (2021) 21:86–92. doi: 10.55460/C9GO-XEUM, 34529811

[7] Stein JA Hepler TC DeBlauw JA Beattie CM Beshirs CD Holte KM . Caffeine maintains vigilance and improves run times during night operations for special forces. Aviat Space Environ Med. (2005) 76:647–54. 16018347

[8] McLellan T Kamimori G Voss D Tate C Smith S. Caffeine effects on physical and cognitive performance during sustained operations. Aviat Space Environ Med. (2007) 78:871–7. 17891897

[9] Tikuisis P Keefe A McLellan T Kamimori G. Caffeine restores engagement speed but not shooting precision following 22 h of active wakefulness. Aviat Space Environ Med. (2004) 75:771–6. 15460628

[10] Irwin C Khalesi S Desbrow B McCartney D. Effects of acute caffeine consumption following sleep loss on cognitive, physical, occupational and driving performance: a systematic review and meta-analysis. Neurosci Biobehav Rev. (2020) 108:877–88. doi: 10.1016/j.neubiorev.2019.12.008, 31837359

[11] Aidman E Balin M Johnson K Jackson S Paech GM Pajcin M . Caffeine may disrupt the impact of real-time drowsiness on cognitive performance: a double-blind, placebo-controlled small-sample study. Sci Rep. (2021) 11:4027. doi: 10.1038/s41598-021-83504-6, 33597580 PMC7889923

[12] Drevon D Fursa SR Malcolm AL. Intercoder reliability and validity of WebPlotDigitizer in extracting graphed data. Behav Modif. (2017) 41:323–39. doi: 10.1177/0145445516673998, 27760807

[13] Borenstein M Hedges LV Higgins JP Rothstein HR. Introduction to Meta-Analysis. Hoboken, NJ, USA: John Wiley & Sons (2021). doi: 10.1002/9781119558378

[14] Chandler J Cumpston M Li T Page MJ Welch V. Cochrane Handbook for Systematic Reviews of Interventions Hob, vol. 4 Wiley (2019). p. 14651858.

[15] Hopkins WG Marshall SW Batterham AM Hanin J. Progressive statistics for studies in sports medicine and exercise science. Med Sci Sports Exerc. (2009) 41:3–13. doi: 10.1249/MSS.0b013e31818cb278, 19092709

[16] Sedgwick P Marston L. How to read a funnel plot in a meta-analysis. BMJ. (2015) 351:h4718. doi: 10.1136/bmj.h4718, 26377337

[17] McLellan TM Caldwell JA Lieberman HR. A review of caffeine’s effects on cognitive, physical and occupational performance. Neurosci Biobehav Rev. (2016) 71:294–312. doi: 10.1016/j.neubiorev.2016.09.001, 27612937

[18] Temple JL Hostler D Martin-Gill C Moore CG Weiss PM Sequeira DJ . Systematic review and Meta-analysis of the effects of caffeine in fatigued shift workers: implications for emergency medical services personnel. Prehosp Emerg Care. (2018) 22:37–46. doi: 10.1080/10903127.2017.1382624, 29324066

[19] Ker K Edwards PJ Felix LM Blackhall K Roberts I. Caffeine for the prevention of injuries and errors in shift workers. Cochrane Database Syst Rev. (2010) 2010:CD008508. doi: 10.1002/14651858.CD008508, 20464765 PMC4160007

[20] Cramer Jenkins E Nunes Soares RA Giansante Barros A Nedelusi A Ferreira de Mello A Marroquin Murcia A . Caffeine, alcohol, and tobacco use in shift-working healthcare professionals: a systematic review. Princ Pract Clin Res. (2026) 11. doi: 10.21801/ppcrj.2025.114.4

[21] Reichenberger DA Lee LJ Hebl J Ayeni AO Robinson II LTD Watkins SL . Sleep and substance use among firefighters: associations with alcohol, caffeine, and nicotine use. Nat Sci Sleep. (2026) 18:605149. doi: 10.2147/NSS.S605149, 42453549 PMC13367355

[22] Clemente-Suarez VJ Robles-Pérez JJ. Acute effects of caffeine supplementation on cortical arousal, anxiety, physiological response and marksmanship in close quarter combat. Ergonomics. (2015) 58:1842–50. doi: 10.1080/00140139.2015.1036790, 25848703

[23] Leenaars CHC Savelyev SA Van der Mierden S Joosten RNJMA Dematteis M Porkka-Heiskanen T . Intracerebral adenosine during sleep deprivation: a meta-analysis and new experimental data. J Circadian Rhythms. (2018) 16:11. doi: 10.5334/jcr.171, 30483348 PMC6196573

[24] Fiani B Zhu L Musch BL Briceno S Andel R Sadeq N . The neurophysiology of caffeine as a central nervous system stimulant and the resultant effects on cognitive function. Cureus. (2021) 13:e15032. doi: 10.7759/cureus.15032, 34150383 PMC8202818

[25] Stepan ME Altmann EM Fenn KM. Caffeine selectively mitigates cognitive deficits caused by sleep deprivation. J Exp Psychol Learn Mem Cogn. (2021) 47:1371–82. doi: 10.1037/xlm0001023, 34014758 PMC12884582

[26] Hudson AN Van Dongen HPA Honn KA. Sleep deprivation, vigilant attention, and brain function: a review. Neuropsychopharmacology. (2020) 45:21–30. doi: 10.1038/s41386-019-0432-6, 31176308 PMC6879580

[27] Cao Y Xie T Ma N. The impairments of sleep loss on core executive functions: general and task-specific effects. Sleep Med Rev. (2025) 84:102163. doi: 10.1016/j.smrv.2025.102163 40946426

[28] Research, I. of M. (US) C. on M. N. "Efficacy of caffeine". In: Caffeine for the Sustainment of Mental Task Performance: Formulations for Military Operations. Washington, DC, USA: National Academies Press (US) (2001). 25057583

[29] Institute of Medicine (Us) Committee on Military Nutrition Research. Caffeine for the Sustainment of Mental Task Performance: Formulations for Military Operations. Washington (DC): National Academies Press (US) (2001). 25057583

[30] Knapik JJ Steelman RA Trone DW Farina EK Lieberman HR. Prevalence of caffeine consumers, daily caffeine consumption, and factors associated with caffeine use among active duty United States military personnel. Nutr J. (2022) 21:22. doi: 10.1186/s12937-022-00774-0, 35421992 PMC9008906

[31] Corry DT Kiefer AW Benedict BA Mihalik JP. Self-reported sleep quality and cognitive performance in special operations forces personnel. Mil Med. (2026) usag299. doi: 10.1093/milmed/usag299, 42378457

[32] Shattuck NL Matsangas P Lawrence-Sidebottom D McClernon CK. Sex differences in U.S. navy sailor well-being, sleep-related behaviors, and psychomotor vigilance performance. Sleep Adv. (2025) 6:zpaf014. doi: 10.1093/sleepadvances/zpaf014, 40365528 PMC12070482

[33] Sterne JAC Sutton AJ Ioannidis JPA Terrin N Jones DR Lau J . Recommendations for examining and interpreting funnel plot asymmetry in meta-analyses of randomised controlled trials. BMJ. (2011) 343:d4002. doi: 10.1136/bmj.d4002, 21784880

[34] Wingelaar-Jagt YQQ Bottenheft C Riedel WJJ Ramaekers JGG. Effects of modafinil and caffeine on night-time vigilance of air force crewmembers: a randomized controlled trial. J Psychopharmacol. (2023) 37:172–80. doi: 10.1177/02698811221142568, 36515156 PMC9912306

[35] Kamimori GH McLellan TM Tate CM Voss DM Niro P Lieberman HR . Caffeine improves reaction time, vigilance and logical reasoning during extended periods with restricted opportunities for sleep. Psychopharmacology. (2015) 232:2031–42. doi: 10.1007/s00213-014-3834-5, 25527035 PMC4432086

