# Supplementary material for: Acute caffeine supplementation as a nutrition-based strategy to mitigate sleep-loss-related cognitive and operational performance impairments in military personnel: a systematic review and meta-analysis
Source: Front Nutr. 2026 Sep 4;13:1893033. doi: 10.3389/fnut.2026.1893033 (PMC13587604; doi:10.3389/fnut.2026.1893033)

Supplementary Appendix

Appendix 1: Search strategy

Table S1. Search strategy of Pubmed

#	Searches
1	((((((((((((Caffeine[MeSH Terms]) OR (caffeine[Title/Abstract])) OR (caffeinated[Title/Abstract])) OR (1,3,7-trimethylxanthine[Title/Abstract])) OR (coffee[Title/Abstract])) OR (tea[Title/Abstract])) OR (cola[Title/Abstract])) OR (soft drink*[Title/Abstract])) OR (carbonated drink*[Title/Abstract])) OR (carbonated beverage*[Title/Abstract])) OR (sport* drink*[Title/Abstract])) OR (sport* beverage*[Title/Abstract])) OR (energy drink*[Title/Abstract])) OR (caffeinated gum[Title/Abstract])
2	((((((((((((((((Sleep Deprivation[MeSH Terms]) OR (sleep deprivation[Title/Abstract])) OR (sleep deprived[Title/Abstract])) OR (sleep loss[Title/Abstract])) OR (sleep restriction[Title/Abstract])) OR (restricted sleep[Title/Abstract])) OR (partial sleep deprivation[Title/Abstract])) OR (total sleep deprivation[Title/Abstract])) OR (total sleep loss[Title/Abstract])) OR (insufficient sleep[Title/Abstract])) OR (sleep deficit[Title/Abstract])) OR (sleep curtail*[Title/Abstract])) OR (wakefulness[Title/Abstract])) OR (sustained wakefulness[Title/Abstract])) OR (prolonged wakefulness[Title/Abstract])) OR (tired*[Title/Abstract])) OR (drowsy[Title/Abstract])) OR (drowsiness[Title/Abstract])
3	#1 AND #2

Table S2. Search strategy of Web of Science

#	Searches
1	TS=("Caffeine" OR "caffeine" OR "caffeinated" OR "1,3,7-trimethylxanthine" OR "coffee" OR "tea" OR "cola" OR "soft drink*" OR "carbonated drink*" OR "carbonated beverage*" OR "sport* drink*" OR "sport* beverage*" OR "energy drink*" OR "caffeinated gum")
2	TS=("sleep deprivation" OR "sleep deprived" OR "sleep loss" OR "sleep restriction" OR "restricted sleep" OR "partial sleep deprivation" OR "total sleep deprivation" OR "total sleep loss" OR "insufficient sleep" OR "sleep deficit" OR "sleep curtail*" OR "wakefulness" OR "sustained wakefulness" OR "prolonged wakefulness" OR tired* OR drowsy OR drowsiness)
3	#1 AND #2

Table S3. Search strategy of Embase

#	Searches
1	'Caffeine'/exp
2	'caffeine':ti,ab,kw OR 'caffeinated':ti,ab,kw OR '1,3,7-trimethylxanthine':ti,ab,kw OR 'coffee':ti,ab,kw OR 'tea':ti,ab,kw OR 'cola':ti,ab,kw OR 'soft drink*':ti,ab,kw OR 'carbonated drink*':ti,ab,kw OR 'carbonated beverage*':ti,ab,kw OR 'sport* drink*':ti,ab,kw OR 'sport* beverage*':ti,ab,kw OR 'energy drink*':ti,ab,kw OR 'caffeinated gum':ti,ab,kw
3	'sleep deprivation'/exp
4	'sleep deprived':ti,ab,kw OR 'sleep loss':ti,ab,kw OR 'sleep restriction':ti,ab,kw OR 'restricted sleep':ti,ab,kw OR 'partial sleep deprivation':ti,ab,kw OR 'total sleep deprivation':ti,ab,kw OR 'total sleep loss':ti,ab,kw OR 'insufficient sleep':ti,ab,kw OR 'sleep deficit':ti,ab,kw OR 'sleep curtail*':ti,ab,kw OR 'wakefulness':ti,ab,kw OR 'sustained wakefulness':ti,ab,kw OR 'prolonged wakefulness':ti,ab,kw OR tired*:ti,ab,kw OR drowsy:ti,ab,kw OR drowsiness:ti,ab,kw
5	#1 OR #2
6	#3 OR #4
7	#5 AND #6

Table S4. Search strategy of SPORTDiscus

#	Searches
1	SU (caffeine) OR "caffeine" OR "caffeinated" OR "1,3,7-trimethylxanthine"OR "coffee" OR "tea" OR "cola"OR "soft drink*" OR "carbonated drink*" OR "carbonated beverage*"OR "sport* drink*" OR "sport* beverage*"OR "energy drink*" OR "caffeinated gum"
2	SU (Sleep Deprivation) OR "sleep deprivation" OR "sleep deprived" OR "sleep loss" OR "sleep restriction" OR "restricted sleep" OR "partial sleep deprivation" OR "total sleep deprivation" OR "total sleep loss" OR "insufficient sleep" OR "sleep deficit" OR "sleep curtail*" OR "wakefulness" OR "sustained wakefulness" OR "prolonged wakefulness" OR tired* OR drowsy OR drowsiness
3	#1 AND #2

Table S5. Search strategy of MEDLINE

#	Searches
1	SU (caffeine) OR "caffeine" OR "caffeinated" OR "1,3,7-trimethylxanthine"OR "coffee" OR "tea" OR "cola"OR "soft drink*" OR "carbonated drink*" OR "carbonated beverage*"OR "sport* drink*" OR "sport* beverage*"OR "energy drink*" OR "caffeinated gum"
2	SU (Sleep Deprivation) OR "sleep deprivation" OR "sleep deprived" OR "sleep loss" OR "sleep restriction" OR "restricted sleep" OR "partial sleep deprivation" OR "total sleep deprivation" OR "total sleep loss" OR "insufficient sleep" OR "sleep deficit" OR "sleep curtail*" OR "wakefulness" OR "sustained wakefulness" OR "prolonged wakefulness" OR tired* OR drowsy OR drowsiness
3	#1 AND #2

Table S6. Search strategy of Cochrane library

#	Searches
1	MeSH descriptor: [Caffeine] explode all trees
2	('caffeine' OR 'caffeinated' OR '1,3,7-trimethylxanthine' OR 'coffee' OR 'tea' OR 'cola' OR 'soft drink*' OR 'carbonated drink*' OR 'carbonated beverage*' OR 'sport* drink*' OR 'sport* beverage*' OR 'energy drink*' OR 'caffeinated gum'):ti,ab,kw
3	MeSH descriptor: [Sleep Deprivation] explode all trees
4	('sleep deprivation' OR 'sleep deprived' OR 'sleep loss' OR 'sleep restriction' OR 'restricted sleep' OR 'partial sleep deprivation' OR 'total sleep deprivation' OR 'total sleep loss' OR 'insufficient sleep' OR 'sleep deficit' OR 'sleep curtail*' OR 'wakefulness' OR 'sustained wakefulness' OR 'prolonged wakefulness' OR tired* OR drowsy OR drowsiness):ti,ab,kw
5	#1 OR #2
6	#3 OR #4
7	#5 AND #6

Appendix 2: Appendix 2: Full-text reports excluded after eligibility assessment

Table S2.1: Full-text articles excluded after eligibility assessment, with reasons

Number	Study	Primary exclusion category	Specific reason
1	Stein et al., 20211	Wrong sleep-loss condition/context	No sleep deprivation or sleep restriction condition.
2	Mills et al., 20012	Wrong population/operational context	Participants were not military personnel or military-related samples, and the task was not military operationally relevant.
3	Gillingham et al., 20043	Wrong sleep-loss condition/context	Examined caffeine effects after fatiguing exercise rather than under sleep deprivation or sleep restriction.
4	Jovanović et al.,20124	Wrong intervention/exposure	No caffeine intervention; the study examined the effect of military training on shooting performance under sleep deprivation.
5	Smith et al., 20175	Wrong intervention/exposure	Investigated sleep restriction and marksmanship without a caffeine intervention.
6	Clemente-Suárez, 20156	Wrong sleep-loss condition/context	Examined acute caffeine effects during stressful close-quarter combat without sleep deprivation or sleep restriction.
7	Wingelaar-Jagt, 20247	Duplicate or companion report	Secondary or companion report of a trial already included; excluded to avoid double counting.
8	Wingelaar-Jagt, 20248	Duplicate or companion report	Duplicate or companion report of an included trial; excluded to avoid double counting.
9	Doan et al., 20069	Insufficient data for effect size calculation	Did not report dispersion data required for meta-analysis, such as standard deviation or standard error.
10	Zhang et al., 201610	Wrong intervention/exposure	Intervention involved coffee/green tea beverages rather than a standardized acute caffeine intervention; caffeine exposure in the green tea group could not be quantified accurately.
11	Tikuisis et al., 200411	Duplicate or companion report	Duplicate record of a study already included.
12	McLellan et al., 201812	Wrong study design	Cross-sectional survey conducted during deployment; no experimental caffeine intervention, comparator, or standardized sleep-loss design.

References:

1. Stein, J. A. *et al.* Caffeine Gum Does Not Improve Marksmanship, Bound Duration, Susceptibility to Enemy Fire, or Cognitive Performance During Tactical Combat Movement Simulation. *Journal of Special Operations Medicine: a Peer Reviewed Journal for SOF Medical Professionals* **21**, 86–92 (2021).
2. Mills, K. C., Spruill, S. E., Kanne, R. W., Parkman, K. M. & Zhang, Y. The Influence of Stimulants, Sedatives, and Fatigue on Tunnel Vision: Risk Factors for Driving and Piloting. *Hum Factors* **43**, 310–327 (2001).
3. Gillingham, R. L., Keefe, A. A. & Tikuisis, P. Acute caffeine intake before and after fatiguing exercise improves target shooting engagement time. *Aviation, space, and environmental medicine* **75**, 865–871 (2004).
4. Jovanović, M., Sporiš, G., Šopar, J., Harasin, D. & Matika, D. The effects of basic military training on shooting tasks in conditions of sleep deprivation. *Kinesiology* **44**, 31–38 (2012).
5. Smith, C. D. *et al.* Sleep restriction and cognitive load affect performance on a simulated marksmanship task. *Journal of Sleep Research* **28**, e12637 (2019).
6. Clemente-Suarez, V. J. & Robles-Pérez, J. J. Acute effects of caffeine supplementation on cortical arousal, anxiety, physiological response and marksmanship in close quarter combat. *Ergonomics* **58**, 1842–1850 (2015).
7. Wingelaar-Jagt, Y. Q., Wingelaar, T. T., Riedel, W. J. & Ramaekers, J. G. Comparison of effects of modafinil and caffeine on fatigue-vulnerable and fatigue-resistant aircrew after a limited period of sleep deprivation. *Frontiers in physiology* **14**, 1303758 (2024).
8. Wingelaar-Jagt, Y. Q. Medicinal fatigue management in military aviation: standard operating procedure or last line of defence? https://doi.org/10.26481/dis.20240926yw (2024) doi:10.26481/dis.20240926yw.
9. Doan, B. K., Hickey, P. A., Lieberman, H. R. & Fischer, J. R. Caffeinated tube food effect on pilot performance during a 9-hour, simulated nighttime U-2 mission. *Aviation, space, and environmental medicine* **77**, 1034–1040 (2006).
10. Zhang, T. *et al.* Effect of coffee and green tea on executive ability and plasma levels of inflammatory factors in soldiers with 48-hour total sleep deprivation. *Int. J. Clin. Exp. Med* **9**, 19354–19362 (2016).
11. Tikuisis, P., Keefe, A. A., McLellan, T. M. & Kamimori, G. Caffeine restores engagement speed but not shooting precision following 22 h of active wakefulness. *Aviation, space, and environmental medicine* **75**, 771–776 (2004).

12. McLellan, T. M., Riviere, L. A., Williams, K. W., McGurk, D. & Lieberman, H. R. Caffeine and energy drink use by combat arms soldiers in Afghanistan as a countermeasure for sleep loss and high operational demands. *Nutritional neuroscience* **22**, 768–777 (2019).

Appendix 3: Leave-one-out sensitivity analysis

Figure . S3.1: Leave-one-out sensitivity analysis: vigilance and response performance

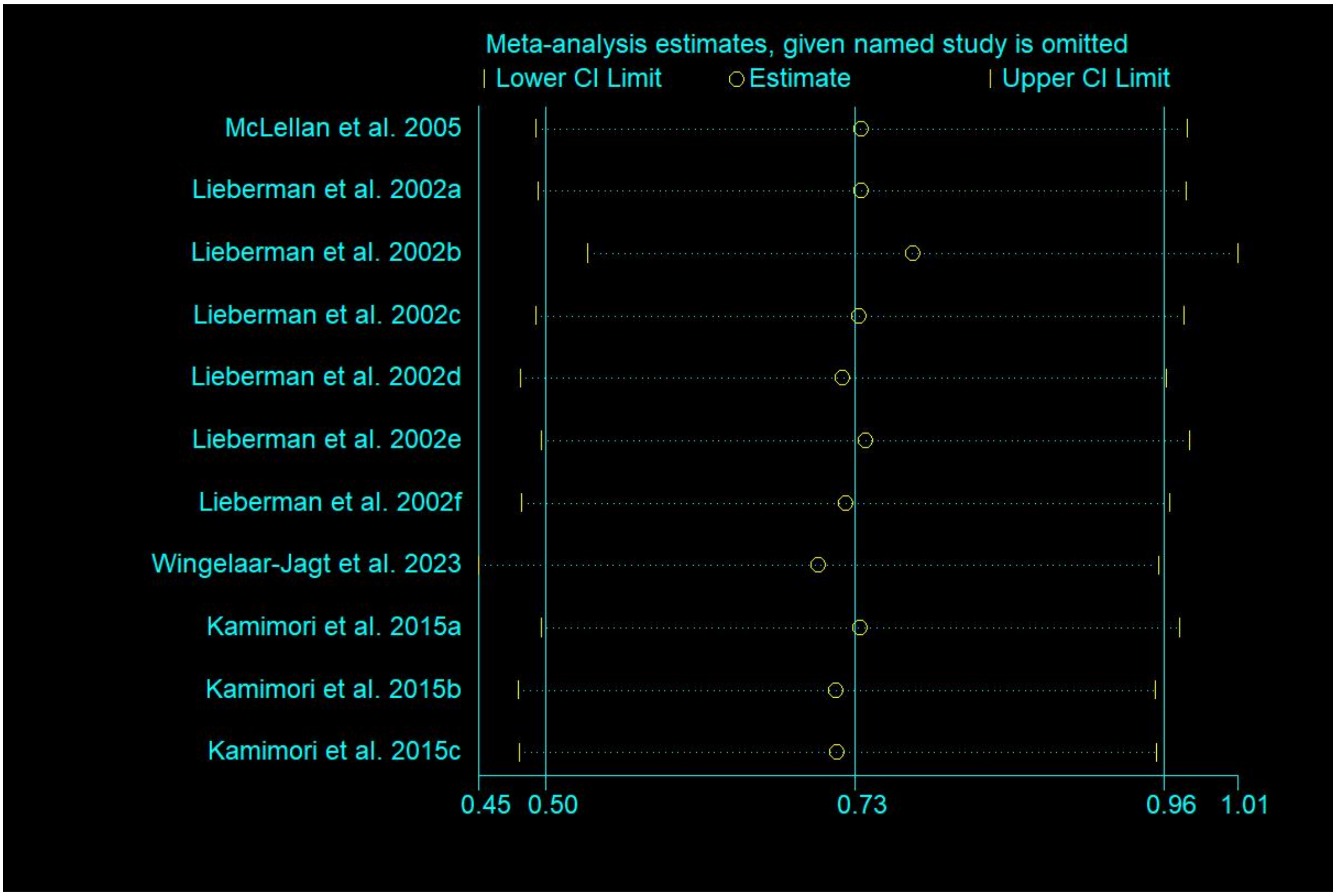

Figure . S3.2: Leave-one-out sensitivity analysis: complex cognitive performance

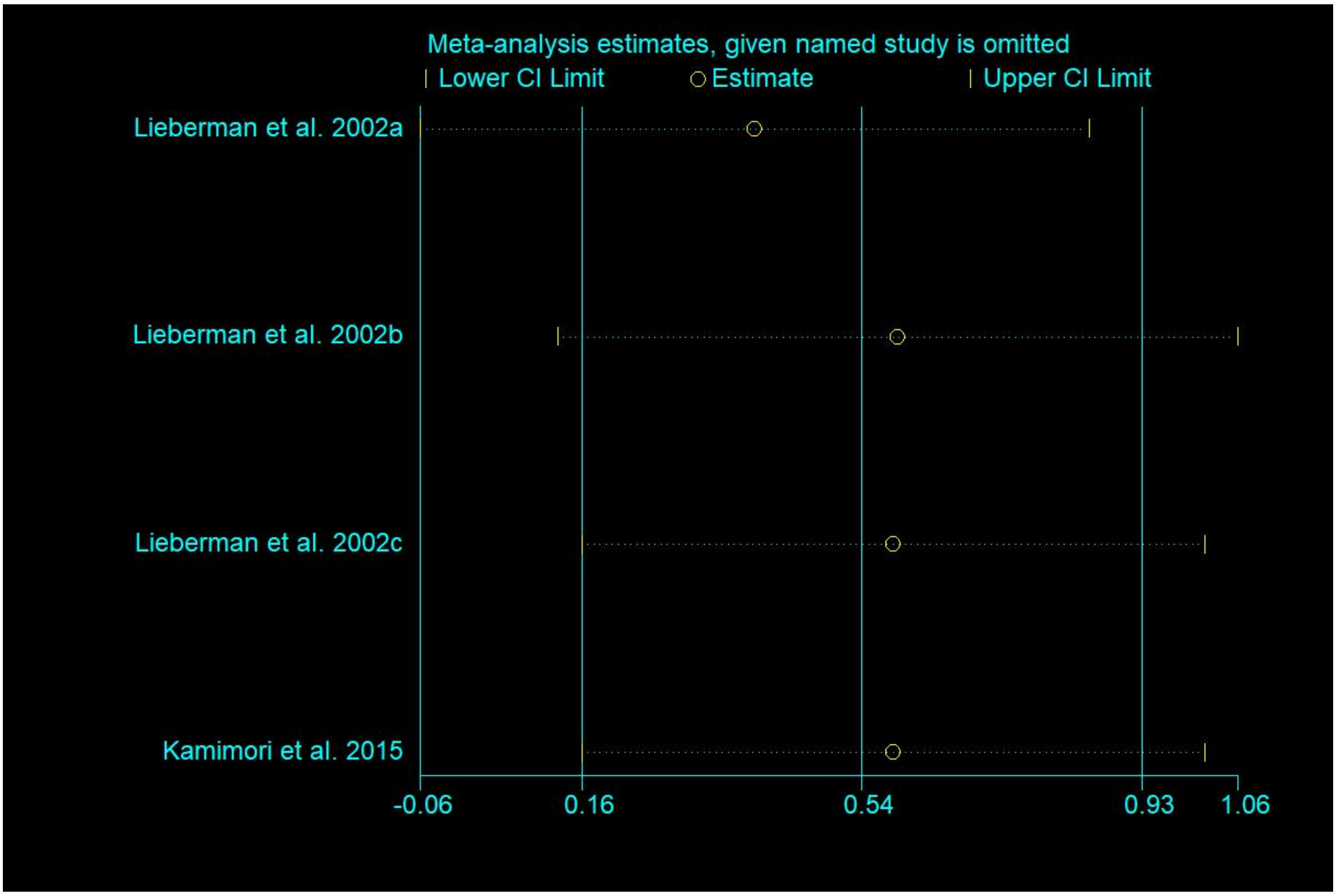

Figure . S3.3: Leave-one-out sensitivity analysis: military task performance

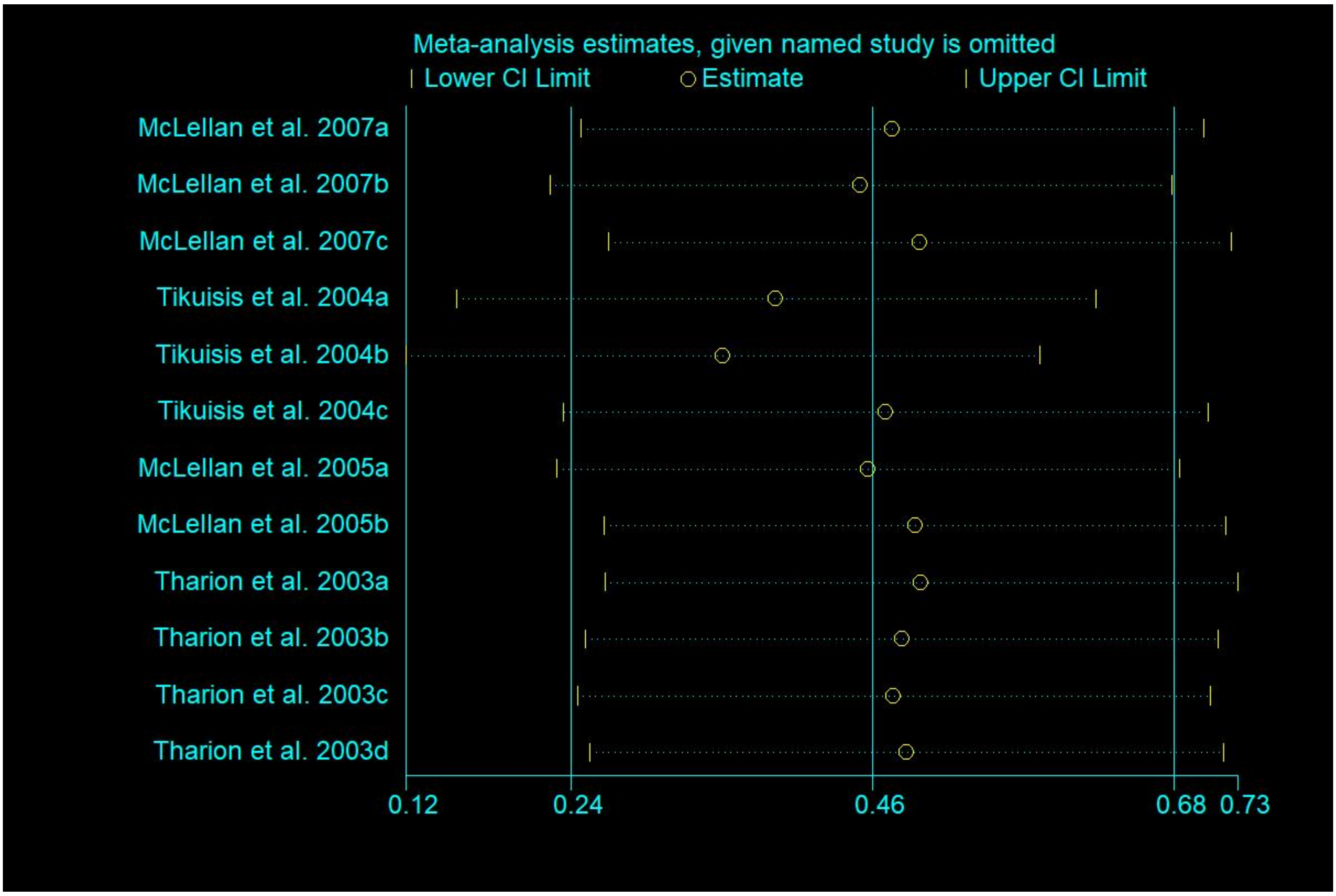

Supplement: Supplementary file 1 [file Data_Sheet_1.PDF]
